# Weed diversity in the reclaimed lands in Middle Egypt

**DOI:** 10.3897/BDJ.13.e154016

**Published:** 2025-08-12

**Authors:** Mahmoud N. Saeed, Ashraf Soliman, Maged S. Ahmad, Shereen M. Korany, Emad Alsherif

**Affiliations:** 1 Botany and Microbiology Department, Faculty of Science, Beni-Suef University, Beni Suef 62521, Egypt Botany and Microbiology Department, Faculty of Science, Beni-Suef University Beni Suef 62521 Egypt; 2 Botany and Microbiology Department, Faculty of Science, Beni-Suef University, Cairo, Egypt Botany and Microbiology Department, Faculty of Science, Beni-Suef University Cairo Egypt; 3 Department of Biology, College of Science, Princess Nourah bint Abdulrahman University, P.O. Box 84428, Riyadh 11671, Saudi Arabia Department of Biology, College of Science, Princess Nourah bint Abdulrahman University, P.O. Box 84428 Riyadh 11671 Saudi Arabia

**Keywords:** weed flora, reclaimed lands, vascular plant diversity, wheat, life form, chorology

## Abstract

More land must be reclaimed for farming due to the expanding population and lack of arable land. Since weeds are considered to be one of the primary causes of agricultural obstacles, the current study sets out to ascertain the weed diversity of the recovered lands in Middle Egypt. We selected eight crop plants, five winter crops and two summer crops — along with five orchards to identify their weed diversity. We found 121 weed species from 91 genera across 26 families — 90 annuals, one biennial and 30 perennials — in the investigated crops and orchards. On the one hand, peach and pomegranate (pome) orchards had the fewest weed species (29 each), while wheat crops had the most (79 species). With six genera, *Euphorbia* was the largest genus and Poaceae had 29 species, the most abundant family. Therophytes were the most prevalent life form amongst weed species, accounting for an average of 72.5%, followed by phanerophytes (5.2%) and geophytes (7.5%). According to the Ward classification of crops and orchards, based on the weeds found there, the planting season had a major influence on the diversity of weeds, followed by the type of plant grown (crop or orchard). The findings demonstrated that the number of years of cultivation had an impact on the weed flora and soil properties in reclaimed fields. Additionally, the old reclaimed areas had higher soil salinity, which reduced the diversity of weeds. Furthermore, xerophytic weed species declined and hydrophytic weed species increased with longer reclamation periods. The current study aims to shed light on the diversity of weeds and their most common species in newly-reclaimed lands in Middle Egypt to facilitate a way to combat them.

## Introduction

Weeds play a variety of roles, by competing for resources like water and nutrients and they pose a significant threat to farmers in terms of crop losses (Alsherif 2020). Additionally, certain weeds are allelopathic, which has a negative impact on yields (Muzell Trezzi et al. 2016). By providing food and shelter to a diverse range of farming species, arable weeds, on the other hand, play a significant role in providing resources and habitat to higher trophic levels (Storkey and Westbury 2007). Weed species have adapted to crops and management practices like ploughing and tillage, which disturb the soil. However, species richness has declined in recent decades due to condensation brought on by chemical fertilisation, herbicide use, high-tolerant cereal plantings and seed-cleaning processes (Roschewitz et al. 2005). Many studies have been carried out in various countries to compare the structure of weed flora with those of earlier studies conducted decades ago to determine the true measure of these intensification factors, as in Germany (Sutcliffe and Kay 2000) and the Czechia (Pys̆ek et al. 2002). Due to their lengthy life cycles, quick germination and growth and abundant seed generation, weeds cause habitat disruption in the areas they invade (Alsherif 2020).

Out of Egypt's approximately one million square kilometres, only 4% is made up of farmed land; the remainder is desert, which is essential to maximise its production potential. Land reclamation transforms deserts into agricultural land and rural villages with the aim of increasing green space to increase agricultural productivity. Egypt is a country with low rainfall; thus, irrigation from the Nile River or groundwater must be supplied to farm the land. Watering these regions, preparing the land for farming, adding manure to boost fertility and constructing the infrastructure of new towns are some ways to do this. Reclaimed lands are species-rich environments because of their diverse habitats, frequent disturbances that produce mosaics of different transitional phases and the introduction of non-native creatures as artificial habitats (Pys̆ek et al. 2002). Reclaimed areas are thought of as a transitional habitat between deserts and ancient farmed lands, when weedy plants replace wild ones due to human activity (Baessler and Klotz 2006). Although numerous studies have been conducted on weeds growing in reclaimed lands in various regions of Egypt (e.g. Abd El-Ghani et al. (2013), Salama et al. (2013), Ibrahim et al. (2022)), the current study aims to shed light on the diversity of weeds and their most common species in reclaimed lands in Middle Egypt to facilitate a way to combat them.

## Material and methods

**The study area**

**Figure 1.**
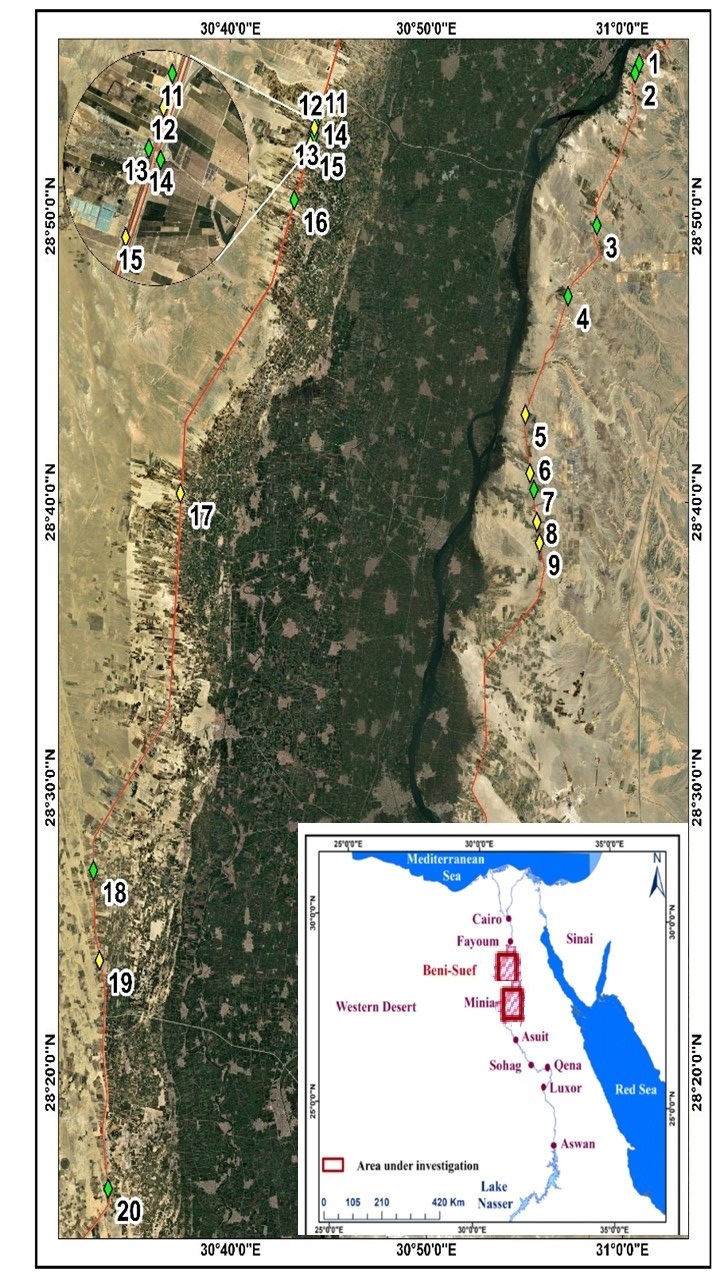
Map showing the different sites in the studied area.

Reclaimed desert lands on the Nile Valley's eastern and western flanks were included in the study area at Middle Egypt (both Beni Suef and Minia Governorates), which is located between 28°55'21.0"N-28°15'11.4"N and 31°00'52.0"E-30°47'16.2"E (Fig. 1). The average monthly temperature of the air varies from 12.4ºC in January to 29.2°C in July. The average humidity ratio is 36% in May to 56% in December, while the average rainfall ranged between zero (from April to October) and 2 mm (from November to March).

**Design of field sampling and data gathering**

Between March 2018 and June 2022, extensive fieldwork was carried out to determine the floristic composition of the research region along the eastern and western banks of the Nile River. The following criteria were used to choose the orchards and crops that were grown: ten locations in each of the study area's two sectors (Fig. 1), including 196 stands (field), were examined using stratified sampling. Five winter crops, two summer crops and five orchards were chosen. The size of the stand (field) varied from site to site, depending on the total area reclaimed and differences in crops and habitats. The presence or absence of specific plant species was recorded in each of the twenty study sites using a number of permanent stands or fields, which were chosen at random to record as much variation in the floristic composition of the agro-ecosystems as possible. The following formula was used to determine each stand's average species frequency (F%): Occurrence rate = n/m X 100, where n is the number of fields containing the species and m is the number of measurement fields there are overall. The local flora (Boulos 1999, Boulos 2000, Boulos 2002, Boulos 2005) was used as the basis for weed identification. At the Beni Suef University Herbarium (BSU), specimens of each species were collected, identified and stored in triplicate. The chorotype of the recorded weeds and life-form classes were determined (Raunkiaer 1934, Zohary 1973).

**Soil analysis**

Soil samples of each crop and orchard were collected at different depths 5, 10 and 15 cm, then mixed well, spread on sheets, left until well air-dried, then gravel and debris removed through sieving by a 2-mm sieve and packed for mechanical and chemical analysis. Soil analysis included: electric conductivity (EC), soil pH, organic matter, grain size distribution, Cl, SO_4_, HCO_3_, CO_3_, Na, K, Ca, Mg and available nitrogen (Bremner 1965).

**Statistical analysis**

To investigate the impact of crop type on weed composition, a presence/absence hierarchical classification study using the Wards (minimal variance) approach and Euclidean distances as a measure of dissimilarity (Ward 1963) was undertaken. The least significant differences (One-Way ANOVA) between the mean values were computed for the soil analysis; when P = 0.05 was reached, terms were deemed significant.

## Results

**Weed composition of different crops and orchards**

**Figure 2.**
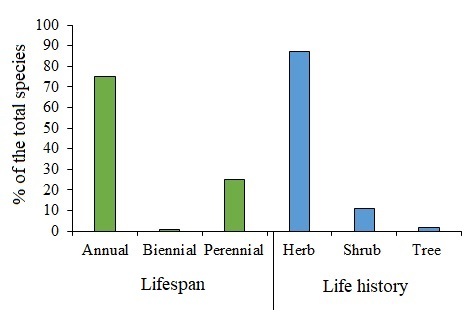
Lifespan and history of the recorded weeds.

Overall, 121 weed species from 91 genera and 26 families were found to be weeds in all farmed orchards and crops on the reclaimed lands under examination. Only one biennial species, *Silybummarianum*, was found in the current study; the remaining 75.3% of the species identified were annual and the remaining 25% were perennial (Fig. 2). Of the documented weeds, 53.7% were winter weeds, 22.3% were summer weeds and just 5.7% were all-year weeds. Desert species, annuals and perennials accounted for 10% of the total species, while marginal and tree species recorded 6% and 2.5%, respectively. In general, the number of species in each family is small, where only four families have 10 species or more, while the other 22 families have less than 10 species (Suppl. material 1). Poaceae was the most species-rich family (29 species), followed by Asteraceae (18 species), Brassicaceae (12 species) and Amaranthaceae (10 species). Ten families were recorded as mono-specific families i.e. each family was represented by only one species (about 38% of all the families that have been documented). The genus *Euphorbia* exhibited the largest species number (6 species), followed by *Amaranthus* L. (4 species), while 75.8% of the recorded genera were represented by only one species (Suppl. material 1).

**Distribution patterns of weed species**

**Figure 3.**
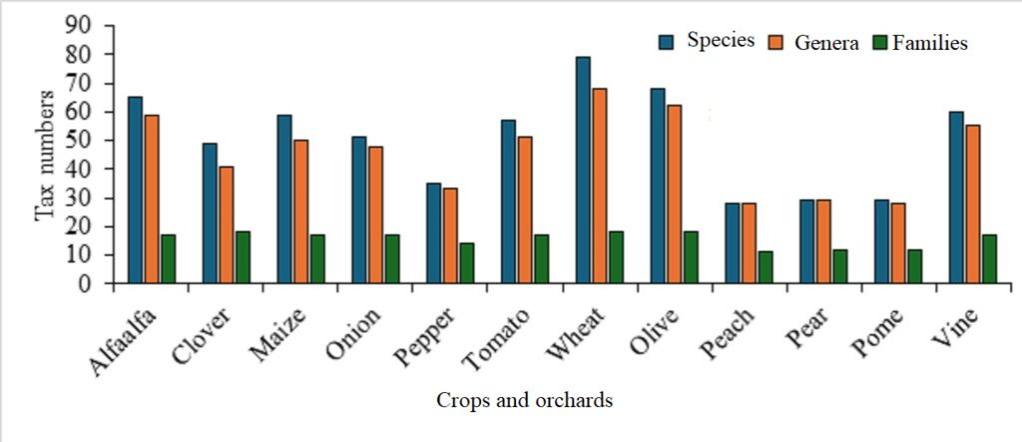
Numbers of species, genera and families of weeds recorded in the studied crops and orchards.

The peach orchard had the fewest weeds species observed, while the wheat crop had the most (Fig. 3). In this study, 28.9% of the observed weed species were occasional species (found in two to five fields) and 31.4% were confined to weeds (found in one field). *Cynodondactylon* and *Cynanchumacutum* were popular year-round weeds, while *Chenopodiummurale* and Malvaparviflora were common winter weeds and *Amaranthusviridis* and *Setariaverticillata* were common summer weeds.

The maximum species number of weeds in inhabited orchards was found in vine orchards. *Phragmitesaustralis* and *Bassiaindica*, two halophytic weeds, were found in olive orchards; also, olives had the highest genera and families. The most common weed species in the crops and orchards under study are listed in Table 1. The most frequent species found in alfalfa, onion, pepper, wheat, olive and peach crops was *Sonchusoleraceous*, whereas the most frequent species found in tomato and pome crops *was Cynodondactylon*. *Portulacaoleracea* was the most frequent species in maize crops, while *Cynanhumacutum* was the most frequent in vine orchard.

**Life forms and chorological affinities**

**Figure 4.**
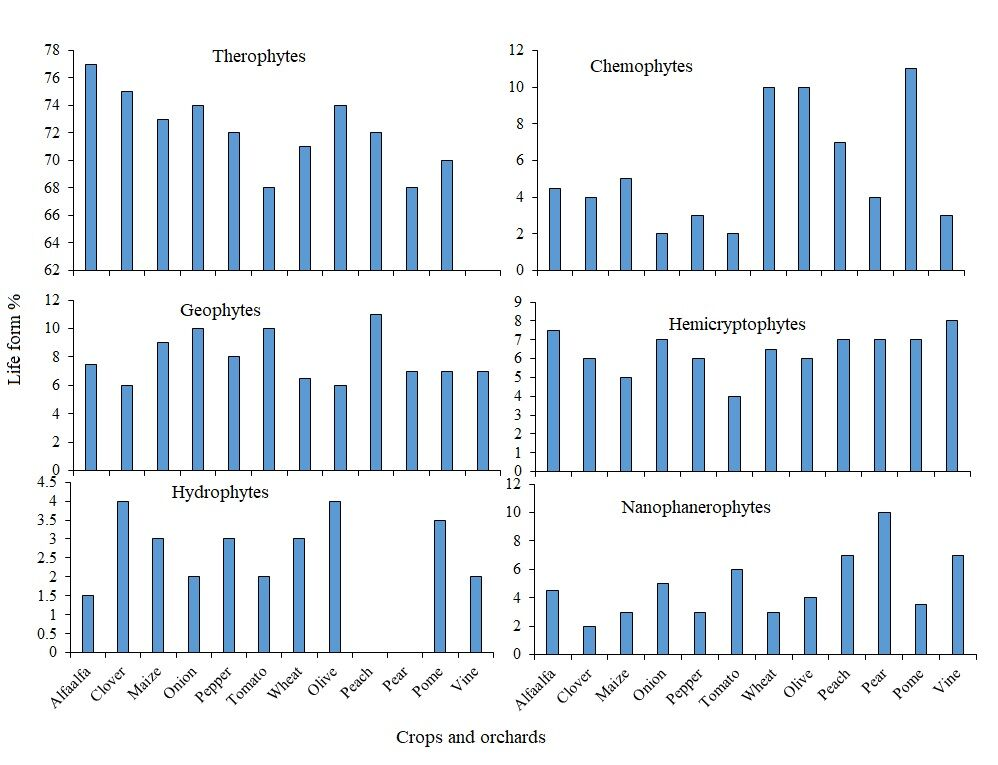
Life forms a percentage of the recorded weeds in the studied crops and orchards.

With ratios ranging from 77% (for clover weeds) to 68% (for pome weeds), therophytes were the predominant life forms for weeds seen in all crops and orchards under study (Fig. 4). With an average of 7.5%, geophytes were the second most prevalent life type; tomato had the highest ratio of geophytes, while clover had the lowest. Phanerophytes had the lowest ratios, ranging from 2.5% in pear orchards to 10% in clover. The chorological investigation revealed that 54 species or 45% of the reported flora were cosmopolitan (widely distributed), pantropical and palaeotropical (Suppl. material 1). Twenty percent (24 species) of the species that were recorded in this study were monoregional. The majority of monoregional species were Saharo-Arabian (6 species) and Mediterranean (7 species). The most prevalent bioregional species in this category are those found in MED+IT (11 species), with SS+SZ recording seven species.

**Effect of crop and orchard types on weed flora**

**Figure 5.**
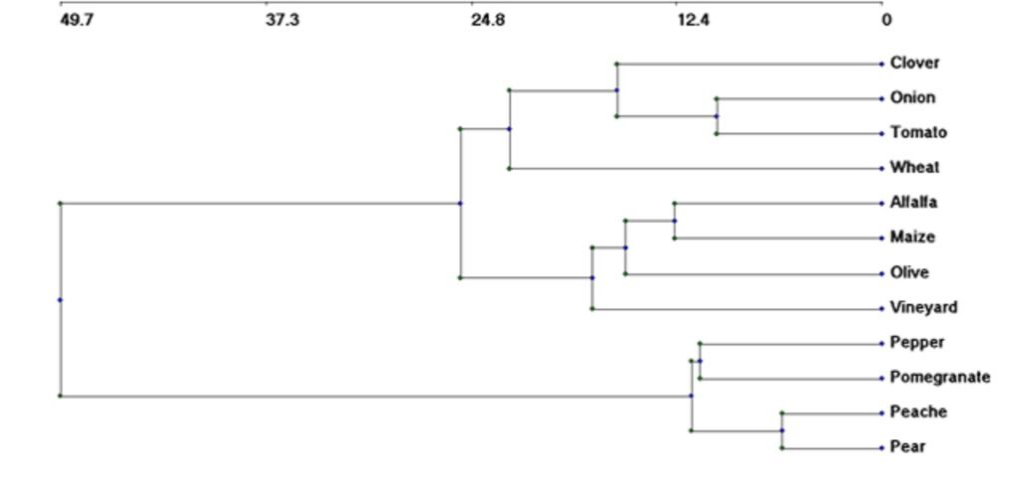
Ward classification of the studied crops and orchards according to their associated weeds.

The hierarchical categorisation yielded a dendrogram (Fig. 5) that distinguished between two main groups of weed flora. The initial group comprised one summer crop (pepper) and three orchards (pear, peach and pomegranate), which were subdivided into two subgroups: one included two orchards (pear and peach) and the other subgroup contained a pomegranate orchard and pepper crop. The second main group contained six crops and two orchards, which were subdivided into two subgroups: one contained weeds of winter crops and the other contained weeds of summer crops with a vineyard and olive orchard. In addition, the dendrogram revealed that onion and tomato crop weeds were floristically more homogeneous than wheat weeds. Jaccard's similarity comparisons revealed that winter crops and summer crops, as well as orchards, have comparable weed compositions (Table 2). In addition, Jaccard similarities between tomato and winter crops exhibited the same values as those of summer crops, with the highest value reported with pepper crops and the lowest value with orchards.

**Effect of reclamation time on soil characters and weed species compositions.**

Two soil samples (for the same crop) were analysed; one had been reclaimed for 12 years and the other for just 8 months, to ascertain the impact of reclamation time on soil chemistry. The following was the result: salinity, accessible nitrogen and organic carbon increased by 35.3%, 110% and 50%, respectively, on the old, reclaimed ground (Table 3). Furthermore, there was a 31.9%, 266%, 36.1% and 33.8% increase in sodium, potassium, calcium and magnesium, respectively, while the amount of SO₄ increased by 22.4% and soil chloride increased by 45.0%.

According to the data, numerous weed species were only found on recently reclaimed land and either totally vanished over time or saw a decline in frequency, such as *Bromuscatharticus*, *Cenchrusechinatus*, *Diplotaxisharra*, *Pulicariaundulata*, *Zygophyllumarabicum*, *Z.coccineum* and *Z.simplex*. In contrast, over a period of time after reclamation, some weeds were recorded that were not there at the beginning, then appeared or their frequency increased with the passage of time, for example, *Lysimachiaarvensis*, *Calendulatripterocarpa*, *Bromusdiandrus*, *BidensPilosa*, *Beta* vulgaris, *Ammimajus*, *Convolvulusarvensis*, *Cuscutacampestris*, *Eragrostisbarrelieri*, *Menthalongifolia*, *Phalarisparadoxa*, *Silybummarianum* and *Urospermumpicroides*.

## Discussion

In the current study, Poaceae, Asteraceae, Brassicaceae and Amaranthaceae were the four largest families; they made up 56.1% of the research area's entire weed flora. Many previous studies reported the former four families were the most prevalent in the reclaimed areas in other parts of Egypt (Shaheen 2002). These families are the most prevalent in the flora of Mediterranean North Africa (Quezel 1978). The recorded weeds were dominated by annuals; this dominance could be attributed to their fast rate of reproduction and the morphological, ecological and genetic flexibility of annuals when there is a lot of disturbance (Alsherif 2020). Furthermore, previously it was stated that annual species were the most common life form in arid and semi-arid regions (da Costa Santos et al. 2007). Due to extensive plantation management techniques such as harrowing, levelling, furrowing, ploughing and subsoiling, which may affect both the life cycles of perennial weeds and vegetative growth structures, the number of perennial weeds decreased. The recorded weeds were dominated by annuals, this seemingly is in agreement with Raunkiaer (1934) who pointed out that their life form is well-adapted to arid climates and they have a core distribution in the desert zones.

*Sonchusoleraceus* was the most widespread and successful weed amongst all crops and orchards. Due totheir vast amplitudes, morphological variety and adaptability, some of the weeds in this study can be interpreted as ubiquitous species, such as *S.oleraceus* and *C.dactylon* (Shaltout and El-Sheikh 2002, Shaltout and El Fahar 2009). Recently, it was reported that *C.dactylon*, a stoloniferous, rhizomatous perennial weed, is amongst the world's most dangerous weeds (Teshirogi et al. 2022). Previously, it was reported that *M.indicus* is an annual noxious weed known to thrive in gardens, orchards, crop fields and canal banks and it was reported that it significantly reduces the yield of crops (Mustafa et al. 2018). It was reported that *Malva* is classified as a noxious weed in Canada and can sprout year-round (Chorbadjian and Kogan 2002). Earlier, it was confirmed that *S.oleraceus* is a weed that is difficult to suppress because of its rapid plant development, intermittent and protracted emergence period and prolific production of extremely dispersive seeds (Peerzada et al. 2019). The habitat choice phenomenon is responsible for the limited range of recorded weeds, such as *C.alopecuroides* and *P.decipiens* (canal bank plants) along canal banks and Tamarix nilotica (halophytic plant) in salinised or waste soils. In addition, the less frequent occurrences of some weed species are due to the habitat choice phenomenon, which causes restricted distribution of some weeds, such as *M.longifolia* and *Oxaliscorniculata* at canal bank habitats.

Significant differences existed in the number and kind of weed species in different crop farmlands, primarily due to crop type and seasonal preference. Furthermore, highly significant correlations were discovered between the olive and grape orchards and the weed flora of the two winter crops, wheat and clover. Weeds in tomato cultivations showed similarities to weeds of summer and winter crops since many tomato fields are grown year-round (winter and summer farming). Each crop has a distinct species composition; most of the species found in each crop are winter weeds, but desert perennials showed notable variations. There may be more desert perennials in olive orchards than other crops because this crop does not require ploughing. As seen by the decline of desert perennials in various crops on the reclaimed areas, canal bank species may have replaced xerophytic species. The large weed population in olive orchards may have been caused by the longer spacing between tree rows and the constant wetness from irrigation. Furthermore, depending on the light circumstances, two different microhabitats were seen in the olive orchard setting: the shaded microhabitat under the olive tree crowns and the sunlit microhabitat in between trees. The microheterogeneity of the environment causes the weed species to form isolated patches. Shade-loving plants, such as *O.corniculata*, *Bidenspilosa* and *Sisymbriumirio*, predominated in the shady parts, whilst other species flourished in the sunny sections of other croplands. Furthermore, the shade produced by the olive orchards keeps the soil moist for a longer period of time than in open areas. Consequently, it permits the growth of species like *Cyperusrotundus*, *Imperatacylindrica* and *P.australis* that are typical of canal banks and damp environments.

The predominant weed life form found in the crops and orchards under study was therophytes, which were followed by chamaephytes. Therophytes' short life cycle, which enables them to tolerate the agro-ecosystem's unpredictable character, is responsible for their domination. Therophytes are usually characterised by a large resource allocation to the reproductive organs and the development of blooms at the start of their lives to guarantee a limited amount of seed production, even in years when the growth season is reduced. The Saharo-Arabian was the predominant chorotype of the flora that was observed; it may have been pure or may have expanded to other regions (mono-, bi- and tri-regional). Saharo-Arabian plants are seen to be good indicators of hot, arid climates since they can survive the exceptionally harsh weather conditions found in these areas, while Mediterranean species are thought to be suggestive of more mesic settings. At least four different floristic elements may be found in Egypt: the Asiatic Irano-Turanian, the African Sudano-Zambezian, the Afro-Asiatic Saharo-Arabian and the Euro-Afro-Asiatic Mediterranean (Alsherif 2020). Pure Mediterranean species were not well represented, such as *Vicialutea* L. and *Papaverrhoeas* L. The degree of salinity and the amount of organic matter are the two main variables that differ significantly between recently reclaimed lands and older reclaimed lands, according to the results of soil analysis. This has a major effect on the quantity and distribution of weed species. These significant differences in certain soil properties, particularly salinity, are highlighted, showing that the soil's salinity rose with time. Farmers must be aware of this and address and correct it immediately to prevent the salt level from rising above what is suitable for producing crops.

## Supplementary Material

DC928011-42C3-52F6-9C42-BB9A3546E39E 10.3897/BDJ.13.e154016.suppl1 Supplementary material 1 Recorded species with their families, chorology, life history and life form Data type Table Brief description The recorded species with their families, chorology, life history and life form. COSM = cosmopolitan, PAL = Palaeotropical, PAN = Pantropical, ES = Euro-Siberian, SZ = Sudano-Zambezian, GC-SZ = Guineo-Congolese-Sudano-Zambezians, ET = Eurimedit.-Turan., MED = Mediterranean, SUD = Sudanian, SS = Saharo-Sindian, SA = Saharo-Arabian TRP = Tropical, N.TRP = Neotropical, IT = Irano-Turanian, ES = Euro-Siberian. Th = Ttherophytes, Hy = Hydrophytes, H = Hemicryptophytes, N. Ph = Nano phanerophytes, M. Ph = Mesophanerophytes. File: oo_1288869.docx https://binary.pensoft.net/file/1288869 Mahmoud N. Saeed, Ashraf Mohamed Soliman, Maged S. Ahmad, Shereen M. Korany, Emad A. Alsherif

## Figures and Tables

**Table 1.** The most frequent weed species in the studied crops and orchards.

**Alfaalfa**	**Clover**	**Maize**
*Sonchusoleraceus* (L.) L.	*Chenopodiastrummurale* (L.) S. Fuentes	*Portulacaoleracea* L.
*Cynodondactylon* (L.) Pers.	*Melilotusindicus* (L.) All.	*Amaranthusviridis* L.
*Cynanchumacutum* L.	*Cichoriumendivia* L.	*Echinochloacolonum* (L.) Link
*Chenopodiastrummurale* (L.) S. Fuentes	*Sonchusoleraceus* (L.) L.	
*Melilotusindicus* (L.) All.	*Malvaparviflora* L.	
*Malvaparviflora* L.	*Phalarisminor* Retz	
**Onion**	**Pepper**	**Tomato**
*Sonchusoleraceus* (L.) L.	*Sonchusoleraceus* (L.) L.	*Cynodondactylon* (L.) Pers.
*Chenopodiastrummurale* (L.) S. Fuentes	*Cynodondactylon* (L.) Pers.	*Chenopodiastrummurale* (L.) S. Fuentes
*Melilotusindicus* (L.) All.	*Chenopodiastrummurale* (L.) S. Fuentes	*Malvaparviflora* L.
*Cynodondactylon* (L.) Pers.	*Bassiaindica* (Wight) A.J. Scott	*Sonchusoleraceus* (L.) L.
*Senecioglaucus* L.	*Amaranthusgraecizans* L.	*Amaranthusviridis* L.
**Wheat**	**Olive**	**Peach**
*Sonchusoleraceus* (L.) L.	*Sonchusoleraceus* (L.) L.	*Sonchusoleraceus* (L.) L.
*Chenopodiastrummurale* (L.) S. Fuentes	*Launaeanudicaulis* (L.) Hook.f.	*Cynodondactylon* (L.) Pers.
*Melilotusindicus* (L.) All.	*Bassiaindica* (Wight) A.J. Scott	*Malvaparviflora* L.
*Malvaparviflora* L.	*Zygophyllumcoccineum* L.	*Chenopodiastrummurale* (L.) S. Fuentes
*Senecioglaucus* L.	*Phragmitesaustralis* (Cav.) Trin. ex Steud.	*Bassiaindica* (Wight) A.J. Scott
**Pear**	**Pome**	**Vine**
*Sonchusoleraceus* (L.) L.	*Cynodondactylon* (L.) Pers.	*Cynanchumacutum* L.
*Cynodondactylon* (L.) Pers.	*Bassiaindica* (Wight) A.J. Scott	*Sonchusoleraceus* (L.) L.
*Malvaparviflora* L.	*Launaeanudicaulis* (L.) Hook.f.	*Cynodondactylon* (L.) Pers.
*Chenopodiastrummurale* (L.) S. Fuentes	*Cynanchumacutum* L.	*Bassiaindica* (Wight) A.J. Scott
*Cynanchumacutum* L.	*Alhagigraecorum* Boiss.	*Phragmitesaustralis* (Cav.) Trin. ex Steud.

**Table 2.** Jaccard similarities of the studied crops and orchards according to their weed composition.

	Clover	Wheat	Alfalfa	Onion	Tomato	Maize	Pepper	Olive	Vineyard	Pomegranate	Peache	Pear
Clover	1											
Wheat	0.54	1.00										
Alfalfa	0.53	0.61	1.00									
Onion	0.61	0.51	0.58	1.00								
Tomato	0.51	0.58	0.56	0.69	1.00							
Maize	0.50	0.52	0.67	0.57	0.51	1.00						
Pepper	0.42	0.41	0.40	0.48	0.53	0.45	1.00					
Olive	0.50	0.55	0.63	0.53	0.52	0.63	0.41	1.00				
Vineyard	0.53	0.58	0.63	0.50	0.62	0.56	0.48	0.58	1.00			
Pomegranate	0.42	0.32	0.32	0.33	0.34	0.35	0.49	0.35	0.37	1.00		
Peach	0.36	0.31	0.37	0.39	0.40	0.34	0.48	0.34	0.38	0.40	1.00	
Pear	0.37	0.35	0.38	0.40	0.41	0.38	0.56	0.37	0.44	0.57	0.65	1.00

**Table 3.** Effect of reclamation time on some soil characters.

Reclamation time		8 months	12 years
pH		7.78	7.67
EC mS/m		12.66	17.14
Anions (eq/l)	CO_3_^--^	-	-
	HCO_3_-	5.5	7.5
	Cl-	68.5	99.5
	SO_4_^--^	52.56	64.35
Cations (eq/l)	Ca^++^	41.5	56.5
	Mg^++^	29.5	39.5
	Na^+^	54.7	72.2
	K^+^	0.86	3.15
	Sand	73.5	71.5
	Silt	23.5	24.5
	Clay	3	4
Texture		Sandy loam	Sandy loam
Available Nitrogen (mg/kg)		60	126
Organic matter %		0.12	0.18

## References

[B12685833] Abd El-Ghani M., Soliman A., Handy R, Bennoba E. (2013). Weed flora in the reclaimed lands along the northern sector of the Nile Valley in Egypt. Turkish Journal of Botany.

[B12685824] Alsherif Emad Ali (2020). Cereal weeds variation in middle Egypt: Role of crop family in weed composition. Saudi Journal of Biological Sciences.

[B12685446] Baessler Cornelia, Klotz Stefan (2006). Effects of changes in agricultural land-use on landscape structure and arable weed vegetation over the last 50 years. Agriculture, Ecosystems & Environment.

[B12686949] Boulos L. (1999). Flora of Egypt, Vol. 1, Azzolaceaea-Oxalidaceae.

[B12686937] Boulos L. (2000). Flora of Egypt, Vol. 2, Geraniaceae-Boraginaceae.

[B12686965] Boulos L. (2002). Flora of Egypt, Vol. 3, Volume three Verbenaceae-Compositae.

[B12686957] Boulos L. (2005). Flora of Egypt, Vol. 4, Monocotyledons.

[B12687042] Bremner J. M., Black C. A. (1965). Method of soil analysis.

[B12685713] Chorbadjian R, Kogan M (2002). Interaction between glyphosate and fluroxypyr improve mallow control. Crop Protection.

[B12687246] da Costa Santos Cristina Mamédio, de Mattos Pimenta Cibele Andrucioli, Nobre Moacyr Roberto Cuce (2007). The PICO strategy for the research question construction and evidence search.. Revista Latino-Americana de Enfermagem.

[B12686917] Ibrahim Lamis, Saleh Amal, Ammar Mohamed, Helmy Mohamed, Abd EL-Hamid Hoda (2022). Weed communities of field crops in the newly reclaimed lands of Suez Canal region, Egypt. Catrina: The International Journal of Environmental Sciences.

[B12687288] Mustafa Ghulam, Ali Abid, Ali Samraiz, Barbanti Lorenzo, Ahmad Mansoor (2018). Evaluation of dominant allelopathic weed through examining the allelopathic effects of four weeds on germination and seedling growth of six crops. Pakistan Journal of Botany.

[B12686862] Muzell Trezzi Michelangelo, Vidal Ribas Antônio, Balbinot Junior Alvadi Antônio, von Hertwig Bittencourt Henrique, da Silva Souza Filho Antonio Pedro (2016). Allelopathy: driving mechanisms governing its activity in agriculture. Journal of Plant Interactions.

[B12685576] Peerzada Arslan Masood, O’Donnell Chris, Adkins Steve (2019). Biology, impact, and management of common sowthistle (*Sonchusoleraceus* L.). Acta Physiologiae Plantarum.

[B12685594] Pys̆ek Petr, Jaros̆ík Vojtĕch, Kuc̆era Tomás̆ (2002). Patterns of invasion in temperate nature reserves. Biological Conservation.

[B12685842] Quezel P. (1978). Analysis of the flora of Mediterranean and Saharan Africa. Annals of the Missouri Botanical Garden.

[B12687000] Raunkiaer C. (1934). The life forms of plants and statistical plant geography.

[B12686899] Roschewitz I, Gabriel D, Tscharntke T, Thies C (2005). The effects of landscape complexity on arable weed species diversity in organic and conventional farming. Journal of Applied Ecology.

[B12686927] Salama Fawzy M., Abd El-Ghani Monier, El Naggar Salah, Aljarroushi Mohamed (2013). Vegetation dynamics and species distribution patterns in the inland desert wadis of South Sinai, Egypt. Ecologia Mediterranea.

[B12685621] Shaheen A. M. (2002). Weed diversity of newly farmed land on the southern border of Egypt (Eastern and Western shores of lake Nasser). Pakistan Journal of Biological Sciences.

[B12685851] Shaltout K. H., El-Sheikh M. A. (2002). Vegetation of the urban habitats in the Nile Delta region, Egypt. Urban Ecosystems.

[B12685630] Shaltout K. H., El Fahar R. A. (2009). Diversity and phenology of weed communities in the Nile Delta region. Journal of Vegetation Science.

[B12686881] Storkey Jonathan, Westbury Duncan B (2007). Managing arable weeds for biodiversity. Pest Management Science.

[B12686908] Sutcliffe Odette L., Kay Quentin O. N. (2000). Changes in the arable flora of central southern England since the 1960s. Biological Conservation.

[B12687278] Teshirogi Koki, Kanno Miho, Shinjo Hitoshi, Uchida Satoshi, Tanaka Ueru (2022). Distribution and dynamics of the *Cynodondactylon* invasion to the cultivated fields of pearl millet in north-central Namibia. Journal of Arid Environments.

[B12685668] Ward Joe H. (1963). Hierarchical grouping to optimize an objective function. Journal of the American Statistical Association.

[B12686992] Zohary M. (1973). Geobotanical foundations of the Middle East.

