# Supplementary material for: Weed diversity in the reclaimed lands in Middle Egypt
Source: Biodivers Data J. 2025 Aug 12;13:e154016. doi: 10.3897/BDJ.13.e154016 (PMC12365670; doi:10.3897/BDJ.13.e154016)
Supplement: Supplementary material 1 — Recorded species with their families, chorology, life history and life form [file bdj-13-e154016-s001.docx]

| **Families** | **Weed species** | **Life history** | | **Chorological group** | **life form** |
| --- | --- | --- | --- | --- | --- |
| Asteraceae Bercht. & J.Presl | *Launaea nudicaulis* (L.) Hook.f. | perennial | herb | SS | He |
| Aizoaceae Martinov | *Trianthema portulacastrum* L. | annual | herb | PAN | Th |
| Amaranthaceae Juss. | *Amaranthus graecizans* L. | annual | herb | MED + IT | Th. |
|  | *Amaranthus hybridus* L. | annual | herb | COSM | Th |
|  | *Amaranthus retroflexus* L. | annual | herb | COSM | Th. |
|  | *Amaranthus viridis* L. | annual | herb | COSM | Th. |
|  | *Bassia indica* (Wight) A.J.Scott | annual | herb | SA + IT | Th. |
|  | *Bassia muricata* (L.) Asch. | annual | herb | SA + IT | Th. |
|  | *Beta vulgaris* L. | annual | herb | MED + IT + ES | Th. |
|  | *Chenopodiastrum murale* (L.) S.Fuentes, Uotila & Borsch | annual | herb | COSM | Th. |
|  | *Chenopodium album* L. | annual | herb | COSM | Th. |
|  | *Haloxylon salicornicum* (Moq.) Bunge ex Boiss. | perennial | shrub | IT | Ch |
| Apiaceae Lindl. | *Ammi majus* L. | annual | herb | MED + IT | Th. |
| Apocynaceae Juss. | *Cynanchum acutum* L. | perennial | herb | MED + IT + ES | Ch. |
|  | *Calotropis procera* (Aiton) W.T.Aiton | perennial | shrub | SS + SZ | M. Ph |
| Asteraceae Bercht. & J.Presl | *Bidens pilosa* L. | annual | herb | PAN | Th. |
|  | *Calendula tripterocarpa* Rupr. | annual | herb | ET | Th. |
|  | *Centaurea scoparia* Sieber ex Spreng. | annual | herb | SS | Ch |
|  | *Cichorium endivia* L. | annual | herb | MED + IT | Th. |
|  | *Erigeron bonariensis* L. | annual | shrub | MED | Th. |
|  | *Galinsoga parviflora* Cav. | annual | herb | COSM | Th. |
|  | *Lactuca serriola* L. | annual | herb | MED | Th. |
|  | *Launaea mucronata* (Forssk.) Muschl. | annual | herb | SA | He |
|  | *Pluchea dioscoridis* (L.) DC. | perennial | shrub | SS + SZ | Ph |
|  | *Pulicaria undulata* (L.) C.A.Mey. | perennial | shrub | SS + SZ | He |
|  | *Reichardia tingitana* (L.) Roth | annual | herb | MED + IT + SA | Th. |
|  | *Senecio glaucus* L. | annual | herb | MED + IT + SA | Th. |
|  | *Silybum marianum* (L.) Gaertn. | biennial | herb | MED + IT + ES | He |
|  | *Sonchus oleraceus* (L.) L. | annual | herb | COSM | Th. |
|  | *Symphyotrichum subulatum* var. squamatum (Spreng.) S.D.Sundb. | annual | herb | PAN | Th. |
|  | *Urospermum picroides* (L.) Scop. ex F.W.Schmidt | annual | herb | MED + IT | Th. |
|  | *Xanthium strumarium* L. | annual | herb | COSM | Th. |
| Brassicaceae Burnett | *Brassica juncea* (L.) Czern. | annual | herb | MED | Th. |
|  | *Brassica nigra* (L.) W.D.J.Koch | annual | herb | COSM | Th. |
|  | *Capsella bursa-pastoris* (L.) Medik. | annual | herb | COSM | Th. |
|  | *Coincya tournefortii* (Gouan) Alcaraz, T.E.Díaz, Rivas Mart. & Sánchez-Gómez | annual | herb | MED + SA | Th. |
|  | *Diplotaxis acris* (Forssk.) Boiss. | annual | herb | SA | He |
|  | *Diplotaxis harra* (Forssk.) Boiss. | perennial | herb | SA | Ch. |
|  | *Farsetia aegyptia* Turra | perennial | shrub | SA + SZ | Ch |
|  | *Lepidium didymum* L. | annual | herb | COSM | Th. |
|  | *Lepidium sativum* L. | annual | herb | MED | Th. |
|  | *Raphanus raphanistrum* L. | annual | herb | IT + ES | Th. |
|  | *Sisymbrium irio* L. | annual | herb | SS | Th. |
|  | *Zilla spinosa* (L.) Prantl | perennial | shrub | SA | Ch. |
| Caryophyllaceae Juss. | *Spergularia marina* (L.) Besser | annual | herb | MED + IT + ES | He |
|  | *Stellaria apetala ucria* | annual | herb | MED + ES | Th. |
| Commelinaceae Mirb. | *Commelina benghalensis* L. | annual | herb | PAL+ SS | Th |
| Convolvulaceae Juss. | *Convolvulus arvensis* L. | perennial | herb | PAL | G. |
|  | *Cuscuta campestris* Yunck. | annual | herb | PAN | Th. |
|  | *Ipomoea biflora* (L.) Pers. . | annual | herb | SA + SZ | Th. |
|  | *Ipomoea carnea* Jacq. | perennial | shrub | PAN | Ch. |
| Cyperaceae Juss. | *Cyperus alopecuroides* Rottb. | perennial | herb | PAN | Hy. |
|  | *Cyperus rotundus* L. | perennial | herb | PAN | G. |
| Euphorbiaceae Juss. | *Euphorbia peplus* L. | annual | herb | COSM | Th. |
|  | *Euphorbia helioscopia* L. | annual | herb | COSM | N. Ph |
|  | *Euphorbia heterophylla* L. | annual | herb | PAN | N. Ph |
|  | *Euphorbia hyssopifolia* L. | annual | herb | TRP | Th. |
|  | *Euphorbia prostrata* Aiton | annual | herb | PAN | Th. |
|  | *Euphorbia serpens* Kunth | annual | herb | SZ | Th. |
| Fabaceae Lindl. | *Alhagi graecorum* Boiss. | perennial | herb | PAL | Th. |
|  | *Medicago polymorpha* L. | annual | herb | COSM | Th. |
|  | *Melilotus indicus* (L.) All. | annual | herb | PAL | Th. |
|  | *Melilotus siculus* (Turra) Steud. | annual | herb | MED + IT | Th. |
|  | *Trifolium resupinatum* L. | annual | herb | MED + IT + ES | Th. |
|  | *Trigonella glabra* subsp. glabra | annual | herb | MED + SS | Th. |
|  | Trigonella laciniata L. | annual | herb | MED + IT + SS | Th. |
|  | *Vachellia nilotica* (L.) P.J.H.Hurter & Mabb. | perennial | tree | SUD | M. Ph |
|  | *Vicia sativa* L. | annual | herb | MED + IT + ES | Th. |
| Lamiaceae Martinov | *Lamium amplexicaule* L. | annual | herb | COSM | Th. |
|  | *Mentha longifolia* (L.) L. | perennial | herb | PAL | Hy. |
| Malvaceae Juss. | *Malva parviflora* L. | annual | herb | MED | Th. |
| Orobanchaceae Vent. | *Orobanche aegyptiaca* Pers. | annual | herb | IT | Th. |
|  | *Orobanche ramosa* L. | annual | herb | COSM | Th. |
| Oxalidaceae R.Br. | *Oxalis corniculata* L. | annual | herb | COSM | G. |
| Papaveraceae Juss. | *Fumaria parviflora* Lam. | annual | herb | MED + IT + ES | Th. |
| Poaceae Barnhart | *Avena fatua* L. | annual | herb | COSM | Th. |
|  | *Bromus catharticus* Vahl | perennial | herb | COSM | Th. |
|  | *Bromus diandrus* Roth | annual | herb | MED | Ch. |
|  | *Cenchrus echinatus* L. | annual | herb | SS + SZ | Th. |
|  | *Cynodon dactylon* (L.) Pers. | perennial | herb | PAN | G. |
|  | *Dactyloctenium aegyptium* (L.) Willd. | annual | herb | PAL | Th. |
|  | *Dichanthium annulatum* (Forssk.) Stapf | perennial | herb | PAL | He |
|  | *Digitaria ciliaris* (Retz.) Koeler | annual | herb | PAL | Th. |
|  | *Dinebra retroflexa* (Vahl) Panz. | annual | herb | SS + SZ | Th. |
|  | *Diplachne fusca* (L.) P.Beauv. ex Roem. & Schult. | perennial | herb | PAL | G. |
|  | *Echinochloa colonum* (L.) Link | annual | herb | PAN | Th. |
|  | *Eleusine indica* (L.) Gaertn. | annual | herb | PAL | Th. |
|  | *Eragrostis barrelieri* Daveau | annual | herb | MED + SS | Th |
|  | *Eragrostis pilosa* (L.) P.Beauv. | annual | herb | PAL + PAN | He |
|  | *Imperata cylindrica* (L.) P.Beauv. | perennial | herb | PAL | G. |
|  | *Leptochloa panicea* (Retz.) Ohwi | annual | herb | PAL | Th. |
|  | *Lolium perenne* L. | perennial | herb | MED + IT | He |
|  | *Moorochloa eruciformis* (Sm.) Veldkamp | annual | herb | PAN | Th. |
|  | *Parapholis incurva* (L.) C.E.Hubb. | annual | herb | MED + IT | Th. |
|  | *Phalaris minor* Retz. | annual | herb | MED + IT | Th. |
|  | *Phalaris paradoxa* L. | annual | herb | COSM | Th. |
|  | *Phragmites australis* (Cav.) Trin. ex Steud. | perennial | herb | PAL | Hy. |
|  | *Poa annua* L. | annual | herb | MED + IT + ES | Th. |
|  | *Polypogon monspeliensis* (L.) Desf. | annual | herb | COSM | Th. |
|  | *Polypogon viridis* (Gouan) Breistr. | perennial | herb | MED + IT | Th. |
|  | *Setaria verticillata* (L.) P.Beauv. | annual | herb | COSM | Th. |
|  | *Setaria viridis* (L.) P.Beauv. | annual | herb | COSM | Th. |
|  | *Urochloa ramosa* (L.) T.Q.Nguyen | annual | herb | SZ | Th |
|  | *Urochloa reptans* (L.) Stapf | annual | herb | PAL | Th. |
| Polygonaceae Juss. | *Persicaria decipiens* (R.Br.) K.L.Wilson . | perennial | herb | PAL | Hy. |
|  | *Portulaca oleracea* L. | annual | herb | COSM | Th. |
|  | *Rumex dentatus* L. | annual | herb | MED + IT | Th. |
|  | *Rumex spinosus* L. | annual | herb | MED | Th. |
|  | *Rumex vesicarius* L. | annual | herb | SS + IT | Th. |
| Primulaceae Batsch ex Borkh. | *Lysimachia arvensis* (L.) U.Manns & Anderb. | annual | herb | COSM | Th. |
|  | *Lysimachia arvensis* var. caerulea (L.) Turland & Bergmeier | annual | herb | COSM | Th. |
| Resedaceae Martinov | *Ochradenus baccatus* Delile | perennial | shrub | MED + IT | Th |
| Solanaceae Juss. | *Hyoscyamus muticus* L. | perennial | shrub | SA+ SZ | He |
|  | *Physalis angulata* L. | annual | herb | PAN | Th |
|  | *Solanum nigrum* L. | annual | shrub | COSM | Th. |
| Tamaricaceae Link | *Tamarix nilotica* (Ehrenb.) Bunge | perennial | Tree | SS + SZ | Ph |
| Urticaceae Juss. | *Urtica urens* L. | annual | herb | MED + IT + ES | Th. |
| Zygophyllaceae R.Br. | *Tribulus terrestris* L. | annual | herb | SS + SZ | Th. |
|  | *Zygophyllum arabicum* (L.) Christenh. & Byng | perennial | shrub | SA | Ch. |
|  | *Zygophyllum coccineum* L. | perennial | shrub | SA | Ch. |
|  | *Zygophyllum simplex* L. | annual | herb | SS | Ch |
